# Photoprotective, Antioxidant and Anti‐Inflammatory Effects of Aged *Punica granatum* Extract: In Vitro and In Vivo Insights

**DOI:** 10.1002/fsn3.70631

**Published:** 2025-08-03

**Authors:** Yuki Ikeda, Mizuho Nasu, Jean‐Yves Bruxer, Rocío Díaz‐Puertas, Jesica Martínez‐Godfrey, Darya Bulbiankova, María Herranz‐López, Vicente Micol, Francisco Javier Álvarez‐Martínez

**Affiliations:** ^1^ Japanese Medical Institute Tokyo Japan; ^2^ Instituto de Investigación, Desarrollo e Innovación en Biotecnología Sanitaria de Elche (IDiBE) Universidad Miguel Hernández (UMH) Elche Spain; ^3^ CIBEROBN (Physiopathology of Obesity and Nutrition CB12/03/30038) Carlos III Health Institute Madrid Spain

**Keywords:** antioxidant, clinical trial, erythema, polyphenol, pomegranate, *Punica granatum*, skin

## Abstract

Ultraviolet (UV) radiation is a primary environmental factor contributing to skin damage, including erythema, hyperpigmentation, and photoaging. This study evaluates the photoprotective, antioxidant, and anti‐inflammatory effects of aged *Punica granatum* extract (APEx), chosen for its richness in polyphenols, to assess its potential as a natural therapeutic agent in skin care. APEx was analyzed for its polyphenolic composition using high‐performance liquid chromatography coupled with mass spectrometry (HPLC‐MS). Its antioxidant capacity was measured via the Trolox Equivalent Antioxidant Capacity (TEAC) assay. Cellular studies on HaCaT keratinocytes and Caco‐2 epithelial cells assessed the extract's antioxidant and anti‐inflammatory effects under stress conditions. A randomized, double‐blind, placebo‐controlled clinical trial involving 60 women evaluated APEx's impact on UV‐induced erythema, melanin content, skin hydration, and lightness. APEx demonstrated high antioxidant capacity (977.08 ± 15.73 mmol Trolox/100 g) and significantly reduced reactive oxygen species and inflammatory cytokine levels in vitro. Clinical findings showed significant reductions in UV‐induced erythema and melanin levels, with concurrent improvements in skin hydration and lightness compared to placebo. No adverse effects were reported during the trial. These findings highlight APEx as a safe and effective agent for mitigating UV‐induced skin damage, offering promising applications in dermatological and cosmetic formulations. Further studies are recommended to explore its molecular mechanisms and effectiveness across diverse populations.

## Graphical abstract

This study evaluates the bioactivity of an aged pomegranate extract (APEx) on human skin. HPLC‐MS identified key compounds: ellagic acid, punicalagin, and urolithin A. In vitro assays confirmed antioxidant, anti‐inflammatory, and low cytotoxic potential. A clinical trial (*n* = 60) assessed APEx under UV exposure, revealing reduced erythema and melanin, and improved hydration and lightness.

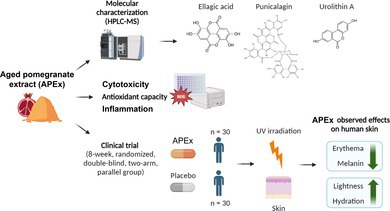

## 1 Introduction

Ultraviolet (UV) radiation is an environmental factor with a great influence on the health and pigmentation of human skin. The level of UV radiation reaching Earth's surface has increased dramatically in recent years due to the depletion of the stratospheric ozone layer (McKenzie et al. 2003). Given that humans are diurnal and exposed to sunlight throughout their lives, UV radiation can create a huge burden of damage to human skin. UV radiation is divided into ultraviolet A (UVA) and ultraviolet B (UVB) radiation (Holick 2016). Although UVB radiation accounts for only 4%–5% of the total UV radiation, it is considered the most active component of sunlight and presents a thousand times greater ability to burn the skin than UVA radiation. Moreover, it penetrates the epidermal layer of the skin and induces direct and indirect adverse biological effects (Kawashima et al. 2018). UVA radiation typically represents > 90% of total UV radiation, and it is known to penetrate the epidermis, thereby promoting the generation of reactive oxygen species (ROS) and damaging underlying structures in the dermis (Battie et al. 2014). Excessive exposure to UV radiation has been directly related to erythema formation (Flo et al. 2017), epidermal hyperplasia (El‐Abaseri et al. 2006), inflammation (Johnson et al. 2013), sunburn (D'Orazio et al. 2013), photoaging (Imokawa and Ishida 2015), immunosuppression (Wang et al. 2019), DNA damage (Mullenders 2018) and increased skin carcinogenesis risk (Liu‐Smith et al. 2017).

Melanin is mainly responsible for human skin pigmentation and is produced by melanocytes in the epidermis in response to UV irradiation through a process called melanogenesis (D'Mello et al. 2016). Under normal physiological conditions, pigmentation has a beneficial effect on the photoprotection of human skin against harmful UV rays (Costin and Hearing 2007). However, excessive production of melanin can cause freckles, pigmented acne scars (Pillaiyar et al. 2017), lentigo solaris (age spots) (Gillbro and Olsson 2011), melasma (Lee et al. 2017), postinflammatory melanoderma (Shenoy and Madan 2020), ephelides pigmentation, and postinflammatory hyperpigmentation (Qian et al. 2020).

Skin pigmentation is influenced by a number of intrinsic factors, including skin type, genetic background, and melanosome amount and dispersal in the skin (Desmedt et al. 2016), and extrinsic factors, including UV radiation exposure, environmental pollution, and drugs (Videira et al. 2013). Avoiding excessive UV‐associated skin pigmentation can be a cosmetic target, especially in Asian countries such as Japan, China, and India where the skin lightening market has increased annually (Pillaiyar et al. 2017). Traditional skin lightening agents employ a melanocyte maturation inhibition mechanism that disrupts the melanogenesis process. Among the most commonly used agents are hydroquinone, aminomercuric chloride, kojic acid, and corticosteroids, although their regulations vary drastically between countries, as some are not legal for use in certain regions. Common adverse effects of these agents include contact dermatitis, permanent melanocyte loss, leukoderma, mutagenic potential, irritation, sensitivity, and itching (Hong et al. 2014; García‐Gavín et al. 2010). The discovery of new natural compounds for skin lightening with fewer or no side effects has attracted the attention of the medicine and cosmetic industry in recent years (Qian et al. 2020).

The objectives of this study were to evaluate the skin protection abilities of a pomegranate extract (APEx) rich in polyphenols. For that purpose, its molecular composition was analyzed and its antioxidant capacity was determined. Subsequently, its antioxidant and anti‐inflammatory capabilities were evaluated in cell models. Finally, a clinical study was conducted to assess the effects of APEx on skin parameters, including erythema severity, melanin content, lightness, water content, and transepidermal water loss, after UV exposure. The study aimed to compare the efficacy of APEx against placebo.

## 2 Materials and Methods

### 2.1 Characterization of Pomegranate Extract

The APEx used in this work is a standardized and fully characterized extract (VIQUA) that was developed and kindly supplied by Axialys Innovations (Annecy, France) and Innovation Labo Sciences Co. Ltd. (Tokyo, Japan). APEx is made from pomegranate fruit‐derived material aged in controlled environmental conditions and is presented in powder form.

#### 2.1.1 Antioxidant Capacity Determination

The antioxidant capacity of APEx was determined using the Trolox Equivalent Antioxidant Capacity (TEAC) assay. In this method, the radical precursor 2,2′‐azinobis(3‐ethylbenzothiazoline)‐6‐sulfonic acid (ABTS) is pre‐treated with potassium persulfate (K_2_S_2_O_8_), and its reduction is directly proportional to the antioxidant capacity of the sample. This reaction leads to a color loss, which is quantified at a wavelength of 734 nm using a spectrophotometer (BioTek Synergy HTX). The results are reported in millimoles of Trolox equivalents per 100 g of dry product.

#### 2.1.2 Molecular Composition Characterization

The high‐performance liquid chromatography (HPLC) system was equipped with A and B pumps (LC‐40D), an autosampler (SIL‐40C), a thermostated oven (CTO‐40C), a DiodeArray detector (SPD‐M30A) and a degasser (DGU‐405) (Shimadzu Scientific Instruments Inc., Japan). LC separation was performed on a Poroshell 120 SB‐C18 column (4.6 × 150 mm, 2.7 μm) (Agilent Technologies Inc., CA, US). The analytical column was kept at 40°C. The mobile phase consisted of acetonitrile (A) and 0.1% formic acid (B). The LC gradient program was: 0–7 min, 5% B; 8–17 min, 18% B; 18–22 min, 50% B; 23–28 min, 90% B; 29–35 min, 5% B. The flow rate was 0.5 mL/min.

HPLC coupled to mass spectrometry (HPLC‐MS) analysis was performed on a Shimadzu LCMS‐8050 triple quadrupole mass spectrometer. The instrument was operated and optimized under positive/negative electrospray and multiple reaction monitoring modes (+ESI MRM and −ESI MRM) using pure standard solutions as well as extract samples. Optimized conditions are as follows: interface voltage, 4.0 kV; interface temperature, 300°C; DL temperature, 300°C; heating block temperature, 400°C; drying gas (N_2_), 10 L/min; nebulizing gas (N_2_), 3 L/min; heating gas (air), 10 L/min; CID Gas (Ar), 230 kPa. All analyses and data processing were completed in Shimadzu LabSolutions V5.109 software.

UV spectra and MS data of the peaks in APEx samples were compared using HPLC grade molecular standards of punicalagin, urolithin A, and ellagic acid (Merck Life Science, Madrid, Spain) or data reported in the literature to identify the extract components.

The total phenolic content of the extracts was determined using the gallic acid equivalent (GAE) method in 96‐well plates. To begin, 10 μL of each sample was combined with 50 μL of Folin–Ciocalteu reagent. After 1 min, 100 μL of a 20% (w/v) sodium carbonate (Na₂CO₃) solution and 840 μL of distilled water were added. The mixture was then incubated in the dark for 30 min. Absorbance was measured at 700 nm using a spectrophotometer (BioTek Synergy HTX, Vermont, USA). A gallic acid standard curve (Sigma‐Aldrich, Missouri, USA) was generated from aqueous solutions of known concentrations. Results were expressed as grams of gallic acid equivalents per 100 g of dry extract (g GAE/100 g dry extract).

### 2.2 Effect of APEx Against UVB‐Induced Cell Damage

#### 2.2.1 Cell Culture

Human skin keratinocyte line HaCaT was procured from Cell Lines Service (Eppelheim, Germany). This cell line was cultured in Dulbecco's Modified Eagle Medium (DMEM, Thermo Fisher Scientific, MA, US) supplemented with Fetal Bovine Serum (FBS, Thermo Fisher Scientific) (10%), penicillin (100 U/mL, Sigma‐Aldrich), and streptomycin (100 μg/mL, Sigma‐Aldrich) at 37°C in a 5% CO_2_/95% air atmosphere in a humidified incubator. Cells were trypsinized every third day, as per the manufacturer's instructions, and seeded in 96‐well plates preceding the assay.

Human colorectal adenocarcinoma immortalized cell line Caco‐2 (American Type Culture Collection) was grown in DMEM containing D‐glucose (4.5 g/L) and supplemented with 10% FBS, 1% non‐essential amino acids (Thermo Fisher Scientific), 1% HEPES (Thermo Fisher Scientific), penicillin (100 U/mL), and streptomycin (100 μg/mL) at 37°C in a humidified atmosphere with 5% CO_2_/95% atmospheric air.

#### 2.2.2 Cell Viability

Firstly, the potential cytotoxicity of APEx in the cells was determined. For that purpose, HaCaT and Caco‐2 cells were seeded at 8000 and 3000 cells/well, respectively, into 96‐well plates. Following a 24‐h pre‐culture period, the medium was removed, and different concentrations of APEx were introduced into each well, where they were incubated for an additional 24 h. The control group consisted of cells that were not exposed to APEx. Cell survival was quantified by nuclear staining using the Hoechst 33342 fluorescent probe (Molecular Probes, Invitrogen/Thermo Fisher Scientific, Waltham, MA, USA). Cells were incubated with the probe (4.5 μM) for 30 min, and the fluorescence was measured using a Cytation 3 Cell Imaging Multimode reader (BioTek, Winooski, VT, USA). The excitation and emission filters used were 377 nm and 447 nm, respectively.

#### 2.2.3 Treatment of Cells

HaCaT cells were seeded at an 8000 cells/well density into two 96‐well plates and incubated with APEx solutions for 5 min. Immediately after, plates were treated with UVB 312 nm light emitted from a UV Bio‐Link BLX crosslinker (Vilber, France) at a dose of 2400 J/m^2^. Negative control wells were covered with aluminum foil to block UVB rays. The following assays were performed 3 h after irradiation.

Caco‐2 cells were seeded at a 3000 cells/well density into two 96‐well plates. Stress conditions were induced by incubating cells in culture medium completed with glucose at 50 mM for a duration of 7 days. Controls were drawn up as follows: low glucose conditions at 5 mM and the untreated high glucose control at 50 mM. Glucose was reintroduced at intervals of about 48 h. APEx was fed into the wells at their respective concentrations, and cells were incubated for another 24 h.

#### 2.2.4 Antioxidant Capacity

Cells were incubated with 30 μM 2′,7′‐dichlorodihydrofluorescein (H2DCFDA, Molecular Probes, Invitrogen/Thermo Fisher Scientific) to monitor ROS generation. H2DCFDA is oxidized by reactive oxygen species to the fluorescent compound 2′,7′‐dichlorodihydrofluorescein (DCF). The DCF signals were normalized to the number of nuclei determined for each well by Hoechst staining. The data were expressed as a percentage of ROS (%) compared to non‐treated and non‐irradiated cells.

#### 2.2.5 Cytokine Analysis

The experiment used the ProcartaPlex Human, NHP, and Canine Mix & Match Panel (Thermo Fisher Scientific Inc., US) to measure the cytokines IL‐1α, IL‐6, IL‐8, and TNFα in cell culture supernatants. All reagents were prepared according to the manufacturer's instructions. Cell culture supernatants were centrifuged to remove particulates, aliquoted, and used immediately. Lyophilized Standard Mix was reconstituted in cell culture medium and serially diluted for the standard curve. Two quality control samples (irradiated vs. non‐irradiated cells) were also included. The assay was conducted in a ProcartaPlex 96‐well flat‐bottom plate (Thermo Fisher Scientific Inc.). The Capture Bead Mix was added to the wells, followed by a bead washing protocol. Standards, controls, samples, and background (cell culture medium) were added to the plate. After incubation and additional washing steps, Biotinylated Detection Antibody Mix and Streptavidin‐PE were sequentially added. Finally, Reading Buffer was added, and the plate was read on a MAGPIX System with xPONENT 4.2 software (Luminex, Austin, TX, US). Cytokine concentrations (pg/mL) were determined based on the measured mean fluorescence intensities (MFIs). The detectable ranges varied for each cytokine: 0.8–3250 pg/mL for IL‐1α, 52–52,800 pg/mL for IL‐6, 3–10,400 pg/mL for IL‐8, and 6–25,200 pg/mL for TNFα.

### 2.3 Human Randomized Controlled Trial

#### 2.3.1 Subjects

The study was designed as an 8‐week double‐blind, two‐arm, parallel group, placebo‐controlled, single‐center randomized clinical trial. Subjects were recruited by phone or e‐mail during 2021 from the city of Tokyo, Japan. All subjects were screened based on inclusion and exclusion criteria and clinically examined, and they were enrolled after providing written informed consent. A total of 96 women were screened during the enrollment period, and 60 of these women (between 25 and 55 years of age) were finally recruited. A simple randomization process was used to assign participants to the APEx treatment group or placebo group. Thirty participants were enrolled in each study group. During the study, 3 volunteers failed to attend one of the study visits, including 2 in the placebo group and 1 in the APEx group, and 57 subjects completed the study.

The clinical trial enrolled healthy nonsmoking women who met the following inclusion criteria: (i) 25 to 55 years of age, (ii) Fitzpatrick skin type I to III, (iii) acceptable contraception method used throughout the study in case of child‐bearing potential, (iv) at least 30 days of stable use for all prescribed medication, (v) willing to provide written informed consent and comply with the trial protocol, (vi) ability to understand the risks/benefits of the protocol, and (vii) availability for the duration of the 8‐week study period. Women were recruited by phone or e‐mail from a clinic within an urban academic medical center. After the subject agreed to participate (signed informed consent), an inclusion criteria checklist was completed, and the subjects were examined in the clinic.

The exclusion criteria for this study were as follows: (i) a current condition and/or disease of the skin that the investigator deemed inappropriate for participation, (ii) women who were nursing, pregnant, or planning to become pregnant during the study, (iii) a preexisting or dormant dermatologic conditions that could interfere with the outcome of the study, (iv) regular consumption or application of antioxidants, that is, vitamin C, CoQ10, and polyphenol extracts, (v) significant history of heart failure, dyslipidemia, diabetes, or uncontrolled hypertension, (vi) risk of food allergy, (vii) extreme skin condition changes caused by menstruation, (viii) current participation in another facial usage study or prior participation in a clinical trial within 4 weeks prior to inclusion into the study, (ix) planned surgeries and/or invasive medical procedures during the course of the study, (x) hormone replacement therapy (HRT) or hormone birth control use initiated less than 3 months prior to study entry or planned for cessation during the study, (xi) facial sunburn or excessive tanned facial skin or lack of willingness to avoid daily facial sun exposure or cease tanning bed or sunless tanning product use for the duration of the study, (xii) currently prescribed or history (within the last 30 days) of oral or topical probiotics or antibiotics, and (xiii) history of psychiatric disorders that may impair the subjects' ability to provide written informed consent.

#### 2.3.2 Study Design and Efficacy Assessment

APEx and placebo capsules were supplied in opaque white plastic bottles that were labeled with an ID number and contained 56 capsules of APEx or placebo. Subjects were asked to take one capsule containing 250 mg of APEx or placebo (dextrin) once a day for 8 weeks and to return the empty bottles to determine adherence.

**FIGURE 1:**
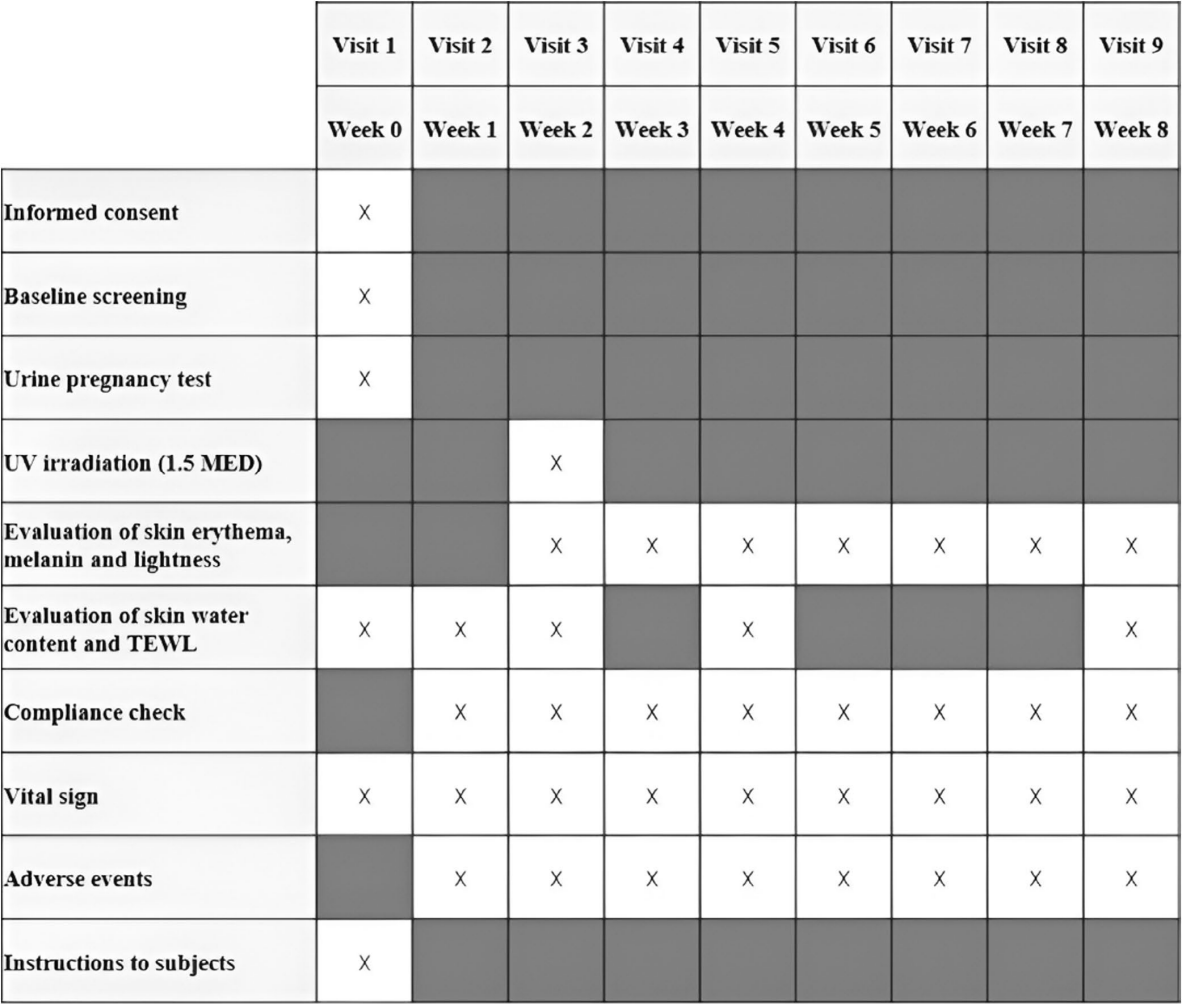
Schematic view of the study design. White squares with an X inside indicate that the event took place.

The efficacy outcome measurements were recorded at baseline and during the study according to the schedule shown in Figure 1. During the first visit, the inside of the subject's upper arm was exposed to solar‐simulated UV radiation using a Xenon Arc solar simulator with Ushio Optical Module × of UVA (23 mW/cm^2^) and UVB (1800 μW/cm^2^) (Ushio Inc., Tokyo, Japan). The skin lightness and minimal erythema dose (MED) of each participant were determined 24 h after irradiation. After 2 weeks of supplementation with the APEx or placebo capsules, skin erythema, lightness, and pigmentation were measured. Then, the inside of the upper arm was irradiated with 1.5 MED of UV. Skin lightness was measured at Weeks 0, 1, 2, 3, 4, 5, and 6 after UV irradiation (Weeks 2, 3, 4, 5, 6, 7, and 8 overall) using a spectrophotometer (Konica Minolta Inc., Osaka, Japan). The water content of the skin was measured using a Corneometer CM 825 (Courage & Khazaka Electronic GmbH, Cologne, Germany) and the TEWL was measured using a TEWAmeter TM 300 (Courage & Khazaka Electronic GmbH, Cologne, Germany) at Weeks 0, 1, 2, 4, and 8 of the study. Water content and TEWL were measured on the subjects' facial skin.

To minimize environmental variability and ensure consistency in skin measurements, all clinical assessments were conducted under controlled temperature (22°C ± 2°C) and relative humidity (45% ± 5%) conditions. The ambient relative humidity and temperature were monitored and recorded during each visit. These controlled conditions were maintained throughout the study to reduce potential confounding effects on skin parameters.

A power analysis was conducted to determine the adequacy of the sample size for detecting statistically significant differences in skin lightness and erythema between the APEx and placebo groups in this 8‐week, double‐blind, placebo‐controlled clinical trial. The primary outcome was the change in skin lightness (L*) after UV‐induced damage and subsequent intervention with either APEx or placebo. Based on prior literature evaluating UV‐protective and antioxidant‐rich supplements, a moderate effect size (Cohen's *d* = 0.6) was assumed. Using a two‐tailed alpha of 0.05 and desired power of 0.80, a minimum of 45 participants (approximately 22–23 per group) would be required to detect a significant group difference using a repeated‐measures design. This calculation is conservative, considering multiple within‐subject measures enhance statistical power. Considering a potential attrition rate of 10%–20%, we decided to recruit 60 participants (30 per group). Recruitment of 60 participants (30 per group) provided a sufficient sample size with over 80% power to detect moderate between‐group differences in skin lightness and erythema over time, assuming similar variability and effect sizes as reported in comparable trials. The repeated‐measures nature of the study (assessments at Weeks 0 through 8) further strengthens the design by increasing sensitivity to treatment effects.

#### 2.3.3 Safety

Subjects had to record any symptoms or reactions observed during the study (information on the risks and benefits of study participation was provided on the consent form). The study team made daily phone calls and email reminders to track subject compliance with the study regimen and identify any reactions or issues. The study coordinator also registered product intake compliance on a form after calling and talking to subjects personally.

#### 2.3.4 Study Ethics

This study was conducted in compliance with the Good Clinical Practices Standards, Nuremberg Code, Declaration of Helsinki, Belmont Report, and associated regulations, and the protocol was approved by the Ethics Review Committee of the Japanese Society of Anti‐Aging Nutrition. Written informed consent was obtained from each patient, and if the patient was unable to read, the patient's legally acceptable representative was present during the entire informed consent process. The subjects' identity and data generated in the study were handled in strict confidence. Access to the raw data was limited to the authorized personnel of the investigator team, ethics committee, sponsor, and regulatory agencies for scheduled monitoring, inspection, and audits. Trial Registration Number: ILCY152020‐S102, November 18, 2020.

### 2.4 Statistical Analysis

Subjects enrolled in the study were randomized in a 1:1 ratio into two groups using a computer‐generated block randomization method, resulting in 30 subjects per group. However, three participants missed one of the study visits: two from the placebo group and one from the APEx group. Ultimately, data from 57 subjects were analyzed, which provided sufficient statistical power to detect significant differences between the APEx group and the placebo group.

All statistical analyses related to the clinical trial data were performed with SPSS for Windows, Version 16.0. (Chicago, SPSS Inc.). Descriptive statistics were used to characterize the sample. Nominal data were analyzed using the chi‐square test, whereas continuous data were analyzed using Pearson's correlation analyses, independent sample 𝑡‐tests, and one‐way analysis of variance (ANOVA), as appropriate. Repeated measures ANOVA was used to assess longitudinal changes over time between groups. For all tests, a *p*‐value < 0.05 was considered statistically significant. Effect sizes (Cohen's *d* for between‐group comparisons and *η* ^2^ for ANOVA) and 95% confidence intervals (CI) were calculated for key outcomes. Data are presented as the means ± standard deviation (SD).

In relation to the cellular assays, the data were represented using GraphPad Prism version 6.0 (GraphPad Software, San Diego, CA, US) and expressed as the mean ± SD. The parameters studied were compared to controls and analyzed using a one‐way ANOVA and Tukey's post hoc test for multiple comparisons. Significant differences between treatments were estimated with a *p*‐value < 0.05.

## 3 Results

### 3.1 APEx Molecular Composition

**FIGURE 2:**
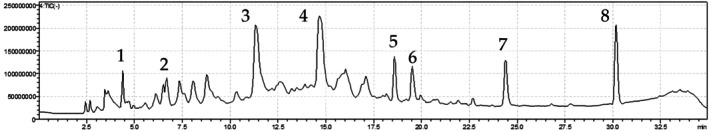
APEx total ion chromatogram obtained by HPLC‐MS. Main detected peaks after chromatogram integration are numbered.

The molecular composition of APEx was analyzed using HPLC‐MS by a method specifically designed for this extract. The total ion chromatogram is shown in Figure 2, and the identified compounds, as well as chromatographic and mass spectral data, are included in Table 1. The MS chromatograms corresponding to each of the peaks described in Table 1 can be observed in Figure S1. Total ion chromatograms of the molecular standards used to identify the main components of APEx can be observed in Figure S2.

**TABLE 1:** Spectral data and identified compounds in APEx using HPLC–MS.

Peak	Retention time (min)	[M‐H]^−^	Peak area	Peak height	S/N	Proposed compound
1	4.394	481	725,328,004	91,866,772	20.15	HHDP‐hexoside
2	6.700	781	1,580,572,797	76,560,369	16.79	Punicalin
3	11.376	1083	5,950,783,627	194,972,002	42.77	Punicalagin α
4	14.704	1083	5,684,212,457	204,998,818	44.97	Punicalagin β
5	18.604	633	1,460,862,081	106,109,738	23.28	Galloyl‐HHDP‐hexose
6	19.524	457	1,639,603,545	95,433,799	20.93	Epigallocatechin gallate
7	24.388	301	1,096,479,280	88,510,174	19.41	Ellagic acid
8	30.169	227	1,970,209,372	194,671,555	42.70	Urolithin A

Molecular standards were used to corroborate the identification of major peaks. Peaks at retention times 11.376 min, 14,704 min, 24.388 min, and 30.169 min were confirmed to correspond to punicalagin α, punicalagin β, ellagic acid, and urolithin A, respectively. Urolithin A was quantified using a standard line (Figure S3) and was determined to make up 5.4% w/w of the extract.

APEx exhibited a total polyphenolic content of 64.68 ± 7.43 g GAE per 100 g of dry extract. Furthermore, APEx demonstrated antioxidant capacity with a TEAC value of 977.080 ± 15.730 mmol Trolox/100 g dry product.

### 3.2 Cellular Assays

**FIGURE 3:**
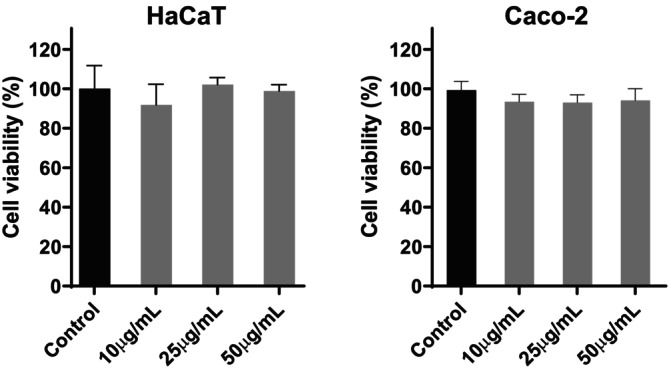
Effect of APEx on cell viability in HaCaT (left graph) and Caco‐2 (right graph) cell models. Untreated controls were included in both cell lines. Statistically significant differences between groups were calculated by using two‐way ANOVA with Dunnett's multiple comparisons correction; however, no statistically significant differences were found.

A Hoechst fluorescent probe was used to investigate the potential cytotoxic effects of APEx on the viability of the external and internal skin barrier of HaCaT and Caco‐2 cells, respectively (Figure 3). Working concentrations were determined and optimized in a previous preliminary study. The assay yielded statistically insignificant data in both cell lines, suggesting the absence of cytotoxicity at the concentrations studied.

**FIGURE 4:**
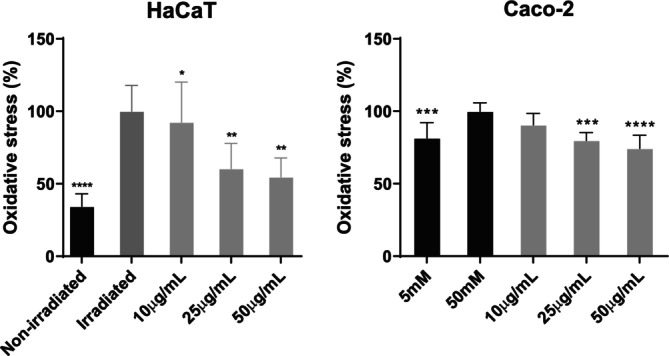
Antioxidant effect of APEx in HaCaT (left graph) and Caco‐2 (right graph) cell models. The level of the H_2_DCF‐DA fluorescent probe was quantified by fluorescence microscopy after irradiation with UVB (HaCaT cells) or after high glucose treatment (Caco‐2). Cells treated with APEx were also exposed to UVB or high glucose, respectively, to assess the protective effect under stress conditions. Non‐treated controls were also included. Statistically significant differences between groups were calculated by using two‐way ANOVA with Dunnett's multiple comparisons correction. *p < 0.05; **p < 0.01; ***p < 0.005; ****p < 0.001.

Subsequently, the ability of APEx to reduce oxidative stress caused by UVB radiation or high glucose concentrations was investigated in HaCaT or Caco‐2 cell lines, respectively, using ROS‐sensitive fluorescent dyes (Figure 4). In the HaCaT cell line, cells treated with APEx showed significantly lower levels of oxidative stress compared to the irradiated control. Furthermore, at concentrations of 25 μg/mL and 50 μg/mL, oxidative stress was reduced by 39.58% and 45.26%, respectively. Similarly, in Caco‐2 cells treated with APEx, there was a notable decrease in oxidative stress compared to the high glucose control. Oxidation levels were significantly reduced by 20.18% and 25.72% at extract concentrations of 25 μg/mL and 50 μg/mL, respectively.

**FIGURE 5:**
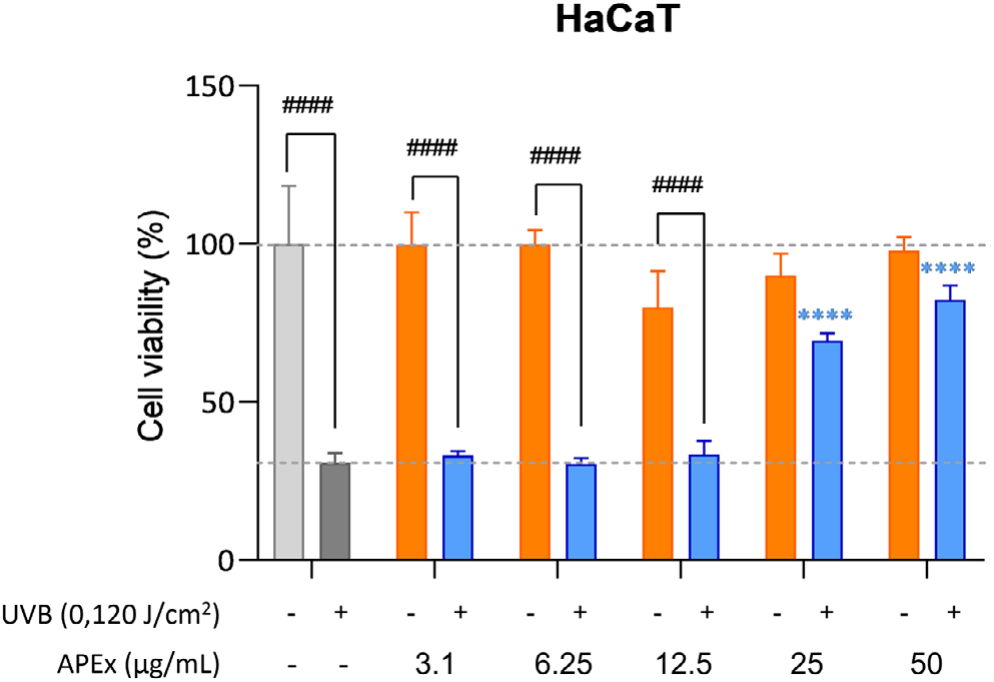
Bar graph of the photoprotection assay of HaCaT keratinocytes with APEx extract at different concentrations after irradiation with UVB (0.120 J/cm^2^). Statistically significant differences between groups were calculated by using two‐way ANOVA with Dunnett's multiple comparisons correction. ****p < 0.001. Samples with the same extract concentration that have significant differences between the irradiated and non‐irradiated group are indicated by a joined line with *p* values #### (*p* ≤ 0.001) according to the Sidak test.

In addition to observing the antioxidant effect of APEx on keratinocytes, another photoprotection test was carried out using a greater range of concentrations to determine the effect of APEx on cell viability after irradiation with UVB. The results obtained correlate with those obtained in Figure 4, since at concentrations of 25 and 50 μg/mL a significant photoprotection is observed that increases the viability of keratinocytes with respect to the irradiated control (Figure 5).

Finally, the extent of the anti‐inflammatory effect was determined by quantification measurements of the four selected cytokine molecules IL‐1α, IL‐6, IL‐8, and TNF‐α, as the most representative pro‐inflammatory cytokines in the inflammatory cellular response. Measurements were performed after stress induction in the cells. In both cell lines, IL‐1α and IL‐8 were quantifiable, whereas IL‐6 and TNF‐α were present at concentrations too low to be accurately quantified. Their experimental levels fell below the detection range of the Luminex xMAP instrument.

**FIGURE 6:**
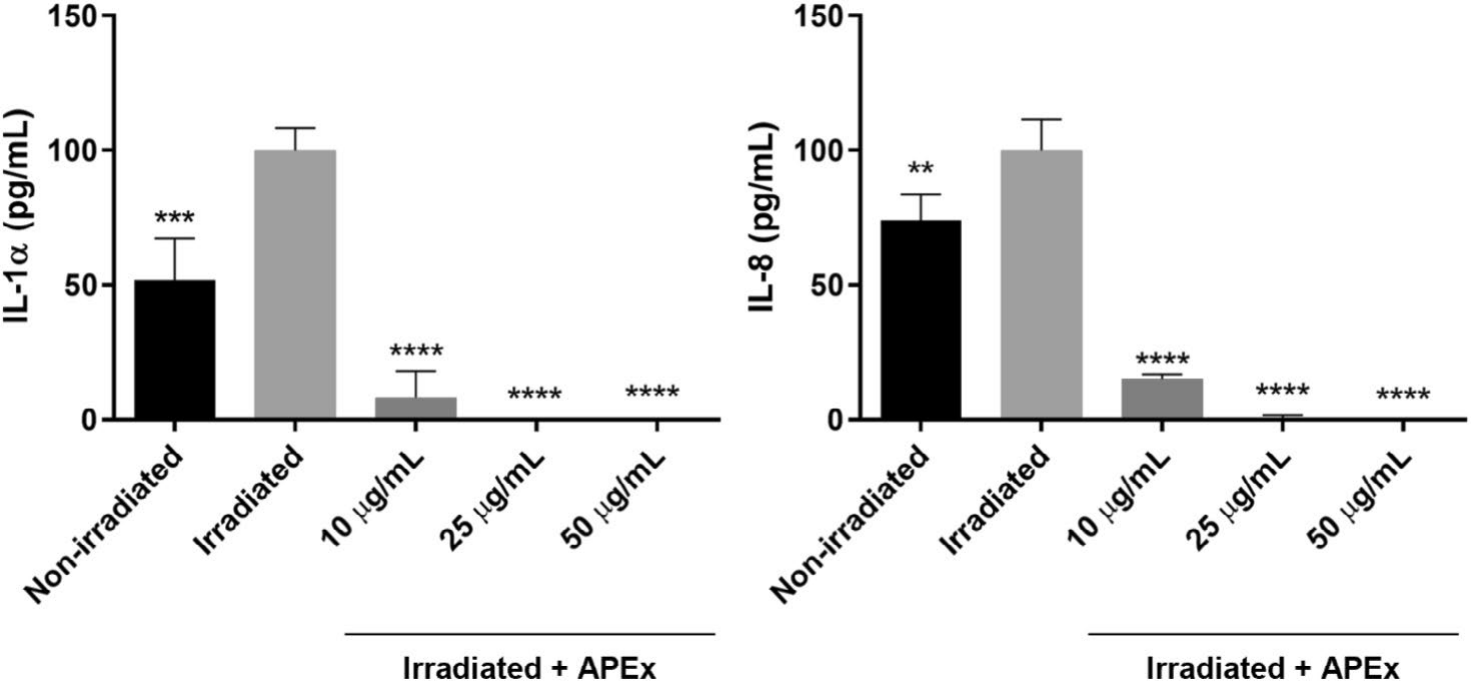
Anti‐inflammatory capacity of APEx measured as IL‐1α (left graph) and IL‐8 (right graph) levels decrease in HaCaT cells. Non‐irradiated and irradiated controls were included. Statistically significant differences between irradiated control and the rest of the samples were calculated by using two‐way ANOVA with Dunnett's multiple comparisons correction **p < 0.01; ***p < 0.005; ****p < 0.001.

In the HaCaT cell line (Figure 6), IL‐1α levels increased dramatically by 48.28% following UVB irradiation compared to the non‐irradiated control. However, treatment with APEx significantly reduced IL‐1α concentrations at all tested doses, reaching values even lower than those observed in the non‐irradiated control. Notably, the 10 μg/mL APEx condition resulted in an immediate 91.95% reduction compared to the irradiated control. Similarly, UVB irradiation caused a significant 26.12% rise in IL‐8 levels relative to the non‐irradiated control. Subsequent treatment with APEx at all concentrations effectively decreased IL‐8 levels, again reaching values below those of the non‐irradiated control. The 10 μg/mL APEx concentration in particular reduced IL‐8 by 85.04% compared to the irradiated control in the absence of the extract.

**FIGURE 7:**
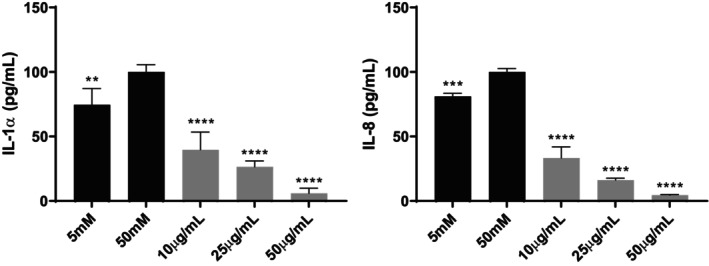
Anti‐inflammatory capacity of APEx measured as IL‐1α (left graph) and IL‐8 (right graph) levels decrease in Caco‐2 cells. Low (5 mM) and high glucose (50 mM) controls were also included. Statistically significant differences between high glucose control and the rest of the samples were calculated by using two‐way ANOVA with Dunnett's multiple comparisons correction **p < 0.01; ***p < 0.005; ****p < 0.001.

In Caco‐2 cells, IL‐1α and IL‐8 levels increased significantly by 25.50% and 13.10%, respectively, following incubation with high glucose (50 mM) compared to the low glucose (5 mM) control, which can be observed in Figure 7. Following treatment with APEx, all concentrations significantly decreased IL‐1α and IL‐8 levels under the various incubation conditions with 50 mM glucose, achieving values lower than those of the 5 mM glucose control. For IL‐1α, APEx concentrations of 10, 25, and 50 μg/mL led to reductions of 60.30%, 73.50%, and 94%, compared to the 50 mM high glucose control. Regarding IL‐8, concentrations of 10, 25, and 50 μg/mL resulted in reductions of 46.12%, 57.93%, and 65.81%, respectively, compared to the 50 mM control.

### 3.3 Human Randomized Controlled Trial

#### 3.3.1 Subjects

**FIGURE 8:**
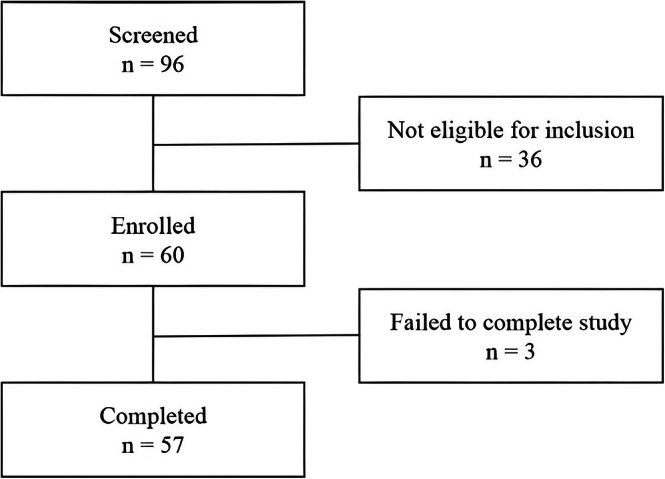
Disposition of the subjects throughout the study.

Figure 8 shows the disposition of the subjects. This clinical trial enrolled 60 subjects. Among them, 3 subjects failed to attend the follow‐up study visit and therefore were excluded from the efficacy analysis. A total of 57 subjects completed the full study.

Table 2 shows the baseline characteristics of the subjects in both groups. Significant baseline differences were not observed between the APEx and placebo groups.

**TABLE 2:** Baseline characteristics of the subjects included in the study.

	Placebo	APEx	*p*
No. of subjects	28	29	
Age (years)	38.5 ± 3.6	37.9 ± 3.4	> 0.05
BMI	24.6 ± 2.5	24.4 ± 2.2	> 0.05
Skin type I	7	8	> 0.05
Skin type II	10	11	> 0.05
Skin type III	11	10	> 0.05
MED (a.u.)	5367.1 ± 316.0	5421.6 ± 354.0	> 0.05
Melanin content (a.u.)	161.6 ± 18.4	158.2 ± 18.4	> 0.05
*L*‐value (a.u.)	61.3 ± 2.1	58.9 ± 2.2	> 0.05
Water content (a.u.)	43.5 ± 3.5	42.7 ± 3.1	> 0.05
TEWL (g/h/m^2^)	9.12 ± 1.05	9.21 ± 1.12	> 0.05

*Note:* *p*‐value > 0.05 indicates no statistically significant differences between groups.

Abbreviations: A.u., arbitrary units; BMI, Body Mass Index; *L*‐value, Lightness value; MED, minimal erythema dose; TEWL, transepidermal water loss.

#### 3.3.2 Efficacy

The effect of consuming APEx or placebo on UV‐induced erythema was evaluated over 6 weeks. Both groups showed an increase in erythema within 24 to 48 h post‐irradiation, with peak levels observed at week 2 (1 week after UV exposure).

Despite the initial rise in erythema values in both groups, a significant difference emerged in the subsequent weeks. At Week 6, the mean change in erythema from baseline (Δ erythema value) was significantly lower in the APEx group (3.7 ± 10.1) compared to the placebo group (34.2 ± 10.5, *p* < 0.05). Significant differences (*p* < 0.05) were observed at each time point, as shown in Table 3. By Week 1, Δ erythema values were 43.5 ± 9.8 in the APEx group, significantly lower than 79.6 ± 10.6 in the placebo group. This trend continued, with Δ erythema values of 23.0 ± 9.7 and 48.1 ± 11.1 in the APEx and placebo groups, respectively, at Week 3.

**TABLE 3:** Changes in the skin erythema, melanin, and lightness values after consumption of APEx or placebo since UV irradiation.

		Period since UV‐irradiation (weeks)					
		1	2	3	4	5	6
Δ Erythema value (a.u.)	Placebo	79.6 ± 10.6^a^	65.3 ± 10.9 ^a^	48.1 ± 11.1 ^a^	42.7 ± 10.8 ^a^	40.3 ± 10.7 ^a^	34.2 ± 10.5 ^a^
	APEx	43.5 ± 9.8^b^	41.1 ± 10.2 ^b^	23.0 ± 9.7 ^b^	21.2 ± 10.3 ^b^	9.3 ± 9.9 ^b^	3.7 ± 10.1 ^b^
Δ Melanin value (a.u.)	Placebo	44.2 ± 6.2 ^a^	46.7 ± 6.4 ^a^	43.9 ± 6.3 ^a^	41.1 ± 6.1 ^a^	38.7 ± 6.2 ^a^	37.3 ± 6.2 ^a^
	APEx	32.0 ± 5.8 ^b^	33.7 ± 5.7 ^b^	31.1 ± 5.8 ^b^	27.6 ± 5.9 ^b^	25.7 ± 5.8 ^b^	23.1 ± 5.8 ^b^
Δ*L*‐value (a.u.)	Placebo	−5.0 ± 1.1 ^a^	−4.6 ± 1.2 ^a^	−4.1 ± 1.1 ^a^	−3.9 ± 1.3 ^a^	−3.6 ± 1.2 ^a^	−3.3 ± 1.1 ^a^
	APEx	−3.5 ± 1.2 ^b^	−3.0 ± 1.4 ^b^	−2.7 ± 1.2 ^b^	−2.5 ± 1.3 ^b^	−2.4 ± 1.1 ^b^	−2.0 ± 1.1 ^b^

*Note:* Significant differences between the placebo and APEx groups at each time point are indicated by different superscript letters (a or b) for each measured parameter. Identical letters denote no significant difference, while different letters indicate statistically significant differences (*p* < 0.05). Superscript letters are applied independently within each parameter (erythema, melanin, and *L*‐value) and are not intended for cross‐parameter comparisons or comparisons across different time points for the same parameter.

Changes in melanin levels also displayed significant differences between the groups (*p* < 0.05) at all time points. At Week 6, the APEx group exhibited a lower Δ melanin value (23.1 ± 5.8) compared to the placebo group (37.3 ± 6.2). Similarly, improvements in skin lightness (Δ*L*‐value) were significantly greater in the APEx group, with values reaching −2.0 ± 1.1 at Week 6, compared to −3.3 ± 1.1 in the placebo group (*p* < 0.05).

These results demonstrate the photoprotective and depigmenting efficacy of APEx, with significant reductions in erythema and melanin levels, as well as improvements in skin lightness, compared to placebo. The consistent and significant differences between groups across all measured parameters suggest that APEx exerts a protective effect against UV‐induced skin damage, likely due to its antioxidant and anti‐inflammatory properties.

Table 4 summarizes the changes in skin water content and TEWL over the course of the study. Measurements were conducted at Weeks 1, 2, 4, and 8 to evaluate the impact of APEx and placebo consumption on these parameters.

**TABLE 4:** Changes in skin water content and TEWL after consumption of APEx or placebo since the study started.

		Study time (weeks)			
		1	2	4	8
Δ Water content (a.u.)	Placebo	1.1 ± 0.7^a^	1.7 ± 0.9 ^a^	2.8 ± 0.8 ^a^	3.5 ± 1.0 ^a^
	APEx	4.9 ± 0.6 ^b^	9.9 ± 1.2 ^b^	12.9 ± 1.5 ^b^	17.2 ± 2.1 ^b^
Δ TEWL (g/h/m^2^)	Placebo	−0.5 ± 0.7 ^a^	−0.9 ± 0.9 ^a^	−1.0 ± 0.8 ^a^	−0.7 ± 0.8 ^a^
	APEx	−0.7 ± 0.6 ^a^	−1.6 ± 0.5 ^b^	−1.8 ± 0.5 ^b^	−2.9 ± 0.7 ^b^

*Note:* Significant differences between the placebo and APEx groups at each time point are indicated by different superscript letters (a or b) for each measured parameter. Identical letters denote no significant difference, while different letters indicate statistically significant differences (*p* < 0.05). Superscript letters are applied independently within each parameter (water content and TEWL) and are not intended for cross‐parameter comparisons or comparisons across different time points for the same parameter.

Significant improvements (*p* < 0.05) in skin hydration were observed in the APEx group compared to placebo at all time points. By Week 1, participants consuming APEx showed a notable increase in skin water content (4.9 ± 0.6 a.u.), significantly higher than the placebo group (1.1 ± 0.7 a.u.). This upward trend continued throughout the study, with water content reaching 17.2 ± 2.1 a.u. by Week 8, compared to 3.5 ± 1.0 a.u. in the placebo group. The sustained rise in skin hydration suggests that APEx effectively enhances moisture retention over time.

In terms of TEWL, a parameter indicative of skin barrier integrity, the APEx group experienced greater reductions compared to placebo, particularly from Week 2 onward. At Week 1, TEWL reductions were not significantly different between the groups (−0.7 ± 0.6 g/h/m^2^ for APEx vs. −0.5 ± 0.7 g/h/m^2^ for placebo, *p* > 0.05). However, by Week 2, the APEx group exhibited a significantly greater decrease (−1.6 ± 0.5 g/h/m^2^) compared to placebo (−0.9 ± 0.9 g/h/m^2^, *p* < 0.05). This trend persisted, with the APEx group showing a TEWL value of −2.9 ± 0.7 g/h/m^2^ at Week 8, indicating a marked improvement in the skin's ability to retain moisture and maintain barrier function.

These results highlight the efficacy of APEx in improving skin hydration and barrier integrity over time. The significant reductions in TEWL observed from Week 2 suggest that the bioactive components in APEx may contribute to enhanced skin barrier repair and protection. In contrast, the placebo group showed only modest changes in both hydration and TEWL, underscoring the superior performance of APEx in promoting skin health.

#### 3.3.3 Safety

No adverse side effects were reported from the consumption of 250 mg of APEx daily for 8 weeks.

## 4 Discussion

The composition analysis revealed that the main components of APEx were punicalagin, urolithin A, and ellagic acid, which are known for their antioxidant and anti‐inflammatory properties (Nguyen‐Ngo et al. 2020; Zhao et al. 2023; D'Amico et al. 2021). The obtained results highlight the high total phenolic content and potent antioxidant capacity of APEx of 977.080 ± 15.730 mmol Trolox/100 g dry product. This value of antioxidant capacity is higher than other extracts of *P. granatum* found in the bibliography, which present values from 439 to 615 mmol Trolox/100 g dry product (Kostka et al. 2020).

The in vitro experiments using epithelial skin (HaCaT) and intestinal (Caco‐2) cell models demonstrated the antioxidant and anti‐inflammatory effects of APEx under stress conditions. Caco‐2 cells are a valuable model for studying the effects of agents on mucosal tissues due to their ability to mimic the intestinal epithelium, their use in barrier function studies, and their relevance to human health (Morresi et al. 2022). The use of Caco‐2 cells as a model to study oxidative stress induced by high glucose levels is well established in scientific research. This model is particularly relevant for investigating the antioxidant and anti‐inflammatory properties of dietary compounds intended for oral consumption. High glucose conditions in Caco‐2 cells are known to mimic the oxidative stress and inflammatory responses seen in metabolic disorders such as diabetes (Morresi et al. 2019). High glucose levels increase oxidative stress markers, such as ROS, and promote the formation of advanced glycation end‐products (AGEs), which are associated with cellular damage and inflammation. Therefore, the use of the Caco‐2 cell model in experiments designed to evaluate the potential antioxidant and anti‐inflammatory properties of orally administered extracts is justified, as it effectively replicates the intestinal environment and oxidative stress conditions relevant to dietary intake (Cianfruglia et al. 2020).

The present findings are consistent with previous studies that have reported the protective effects of pomegranate extracts on epithelial cells. For example, Zaid et al. (2007) evaluated the effect of pomegranate extract on HaCaT cells after UVB exposure. They found that the extract prevented the decrease in cell viability induced by UVB exposure, reduced intracellular glutathione levels, and inhibited the increase in lipid peroxidation (Zaid et al. 2007). In another study by Mastrogiovanni et al. (2019), pomegranate extracts were observed to decrease the expression levels of the inflammatory cytokines IL‐1α, IL‐6, and CXCL8 in TNF‐stimulated Caco‐2 cells. In terms of their mechanism of action, polyphenols, such as those present in pomegranate extracts, are acknowledged for their capacity to dampen inflammatory reactions by inhibiting the nuclear factor kappa B (NF‐κB) and mitogen‐activated protein kinase (MAPK) signaling pathways (Mastrogiovanni et al. 2019).

The results of the present study show that, upon exposure to UV‐radiation, 8 weeks of oral consumption of APEx led to a decrease in erythema value, melanin content, and TEWL, and an increase in skin lightness and water content compared with the control group. Previous studies have supported the efficacy of oral plant‐based extracts supplementation in minimizing pigmentation and human skin damage after exposure to solar radiation, including UV‐induced erythema, early aging, inflammation, and irradiation‐induced cancer (Reuter et al. 2010; Pérez‐Sánchez et al. 2018). The ability of plant polyphenols to reduce erythema has been previously described (Nobile et al. 2016; Pérez‐Sánchez et al. 2014). Topical application of green tea (Camouse et al. 2009), pistachio seeds and peel (Martorana et al. 2013), *Epilobium angustifolium* (Ruszová et al. 2013) extracts rich in polyphenols showed in vivo skin photoprotection after UV irradiation.

UV irradiation can lead to DNA damage due to the direct absorption of UVB radiation by DNA molecules (Rastogi et al. 2014) or indirect ROS production, which stimulates inflammatory processes, such as the activation of transcription factors that regulate the proteolytic degradation of the skin extracellular matrix (Pillai et al. 2005). Pomegranate‐derived products have shown protective abilities against UVB‐mediated DNA damage in human reconstituted skin based on their strong antioxidant activity (Afaq et al. 2009). Furthermore, pomegranate concentrate has been shown to inhibit pigmentation via the inhibition of tyrosinase activity on the skin of brownish guinea pigs receiving UV irradiation (Henning et al. 2019).

Ellagic acid has been widely used as a supplement in cosmetics based on its reported anti‐inflammatory and antioxidant properties (Rosillo et al. 2012). Urolithins are metabolites produced by the breakdown of ellagitannins and ellagic acid by intestinal microbiota, and they are better absorbed than their precursors and can contribute significantly to the beneficial properties attributed to ellagitannins and ellagic acid (Selma et al. 2017). However, great interindividual variability is observed in these health effects because different phenotypes are observed depending on the type of urolithin that the microbiota is capable of producing (Tomás‐Barberán et al. 2014). Direct supplementation with products containing urolithins can overcome the limitations of dietary exposure and gut microbiome variability (Singh et al. 2022). Urolithin A is metabolized after intake into 3‐O‐glucuronide, urolithin A 3‐sulfate, and urolithin A‐sulfate glucuronide (Lin et al. 2023). Urolithin A is capable of increasing the expression of genes related to mitochondrial biogenesis and oxidative phosphorylation (Esselun et al. 2021), in addition to exhibiting a high anti‐inflammatory capacity (Toney et al. 2021). These properties of urolithin A could help increase the resistance of skin cells and cell viability against oxidative stress such as UVB.

The photoprotective and depigmenting effects observed for APEx may be explained by the modulation of specific intracellular signaling pathways involved in oxidative stress and melanogenesis. Polyphenols such as punicalagin and ellagic acid have been shown to inhibit UV‐induced activation of the MAPK pathway, particularly by reducing the phosphorylation of p38 and JNK, which play a role in the upregulation of matrix metalloproteinases and photoaging processes (Afaq et al. 2009; Bae et al. 2010). Moreover, ellagic acid can suppress the expression of tyrosinase and MITF, two central regulators of melanin biosynthesis, by interfering with the cAMP/PKA/CREB pathway (Bae et al. 2010). In parallel, urolithin A has been reported to activate the AMPK‐SIRT1‐PGC1α axis, promoting mitochondrial function, energy homeostasis, and cellular resistance to oxidative stress (D'Amico et al. 2021).

Although this study primarily evaluates the short‐term effects of APEx, the long‐term safety and stability of its main polyphenols have been extensively documented. Punicalagins and ellagic acid are relatively stable compounds under controlled storage conditions and retain their antioxidant properties over time when protected from moisture and excessive heat (Kostka et al. 2020). Moreover, urolithin A, one of the key metabolites detected in APEx, has shown good chemical stability and safety in repeated‐dose human studies (D'Amico et al. 2021). Previous research has demonstrated that ellagic acid and punicalagin do not accumulate in tissues and are excreted efficiently, reducing the risk of long‐term toxicity (Nguyen‐Ngo et al. 2020; Rosillo et al. 2012). Furthermore, clinical studies involving up to 12‐week oral supplementation of pomegranate‐derived extracts or ellagitannin‐rich formulations have reported no adverse effects (Afaq et al. 2009; Henning et al. 2019). While our study confirms safety over an 8‐week period, these findings, along with the GRAS (Generally Recognized as Safe) status of pomegranate components, support the safe long‐term use of APEx in nutraceutical and cosmetic applications.

There are multiple studies that demonstrate the skin protective and lightening effects of plant extracts rich in polyphenols both in vitro (Nobile et al. 2016; Smeriglio et al. 2019) and in vivo (Pérez‐Sánchez et al. 2014). In addition, there are studies that demonstrate the skin lightening capacity of mixtures of polyphenolic plant extracts, including those of pomegranate, osmanthus, and olive, rich in punicalagin, verbascoside, and hydroxytyrosol, respectively (Wang et al. 2021). Pomegranate extracts are rich in polyphenols with demonstrated skin‐protective capacity, particularly ellagic acid (Bae et al. 2010), punicalagins (Houston et al. 2017) and metabolites generated by their conversion such as urolithin A, which can also display significant skin health related activities (Wang et al. 2017). Through their capacity to scavenge free radicals and modulate various signaling pathways involved in melanogenesis, these polyphenols contribute to skin health by reducing inflammation, preventing photoaging, and promoting an even skin tone (Pérez‐Sánchez et al. 2018; Sánchez‐Marzo et al. 2020). Clinical trials in humans have supported the antimelanogenic and skin lightening capacity of plant‐based agents. Some examples are the skin lightening characterization of *Rosa gallica* petal extract (Song et al. 2020), *Sophora flavescens* root extract (Shin et al. 2013), *Pyrus communis* fruit extract (Khiljee and Akhtar 2019) or *Prosopis cineraria* bark extract (Mohammad et al. 2018). The mechanisms of action identified in these agents include the activation of mitogen‐activated protein kinase, inhibition of the tyrosinase enzyme, and downregulation of melanosome formation and transport at mRNA and protein levels in keratinocytes. Furthermore, protective agents ingested orally have certain advantages over classic agents for topical application because the latter may not be distributed evenly over the entire skin, may have skin absorption problems, or may be negatively affected by washing, perspiration, or rubbing with other surfaces (Sondenheimer and Krutmann 2018).

These findings suggest that APEx can be considered an effective agent for skin protection against solar damage and hyperpigmentation. However, the study's limitations, including sample size, trial duration, and interindividual variability, should be considered. Future studies should investigate the underlying molecular mechanisms and evaluate the efficacy of APEx in different populations for longer periods.

## 5 Conclusions

In summary, this study evaluated the protective effects of APEx against UV‐induced skin damage in a randomized, controlled clinical trial. The results obtained indicate that APEx, rich in polyphenols, exhibits significant antioxidant and anti‐inflammatory capabilities. In cellular models, APEx significantly reduced the generation of reactive oxygen species and the release of inflammatory cytokines under induced stress conditions. Furthermore, the clinical trial demonstrated that APEx application decreased the severity of erythema and melanin content, and improved skin hydration and lightness compared to the placebo group. In conclusion, APEx holds significant potential as an antioxidant ingredient in dermatological and cosmetic formulations aimed at skin protection and care.

## Author Contributions

**Yuki Ikeda:** conceptualization (equal), data curation (equal), formal analysis (equal), methodology (equal). **Mizuho Nasu:** investigation (equal), project administration (equal), validation (equal). **Jean‐Yves Bruxer:** conceptualization (equal), methodology (equal), supervision (equal). **Rocío Díaz‐Puertas:** investigation (equal), writing – original draft (equal). **Jesica Martínez‐Godfrey:** investigation (equal), software (equal). **Darya Bulbiankova:** investigation (equal), writing – original draft (equal). **María Herranz‐López:** methodology (equal), writing – review and editing (equal). **Vicente Micol:** data curation (equal), writing – original draft (equal), writing – review and editing (equal). **Francisco Javier Álvarez‐Martínez:** data curation (equal), software (equal), writing – original draft (equal), writing – review and editing (lead).

## Ethics Statement

All procedures performed in studies involving human participants were in accordance with the ethical standards of the institutional and/or national research committee and with the 1964 Helsinki Declaration and its later amendments or comparable ethical standards. The study was approved by the Japanese Society of Anti‐Aging Nutrition. Trial Registration Number: ILCY152020‐S102, November 18, 2020.

## Consent

Informed consent was obtained from all subjects involved in the study.

## Conflicts of Interest

Innovation Labo Sciences Co. Ltd. partially funded the clinical trial and provided the test product samples. However, the sponsor had no role in the design or conduct of the study, the data analysis, or the decision to publish. These tasks and the preparation of the manuscript were performed by F.J.Á.‐M., R.D.‐P., and V.M., who are part of the Miguel Hernández University of Elche, a public and completely impartial university. Although Innovation Labo Sciences Co. Ltd. was allowed to review the manuscript and suggest changes, the final decision on content remained exclusively with the corresponding author. Dr. V.M. serves as the guarantor for this article, taking full responsibility for the integrity of the work in its entirety.

## Supporting information


**Figure S1:** fsn370631‐sup‐0001‐FiguresS1‐S3.docx.

## Data Availability

The datasets generated during and/or analyzed during the current study are available from the corresponding author on reasonable request.
