# Supplementary material for: Photoprotective, Antioxidant and Anti‐Inflammatory Effects of Aged Punica granatum Extract: In Vitro and In Vivo Insights
Source: Food Sci Nutr. 2025 Aug 3;13(8):e70631. doi: 10.1002/fsn3.70631 (PMC12318356; doi:10.1002/fsn3.70631)
Supplement: Supplementary file 1 — Figure S1: fsn370631‐sup‐0001‐FiguresS1‐S3.docx. [file FSN3-13-e70631-s001.docx]

**Supplementary material:**


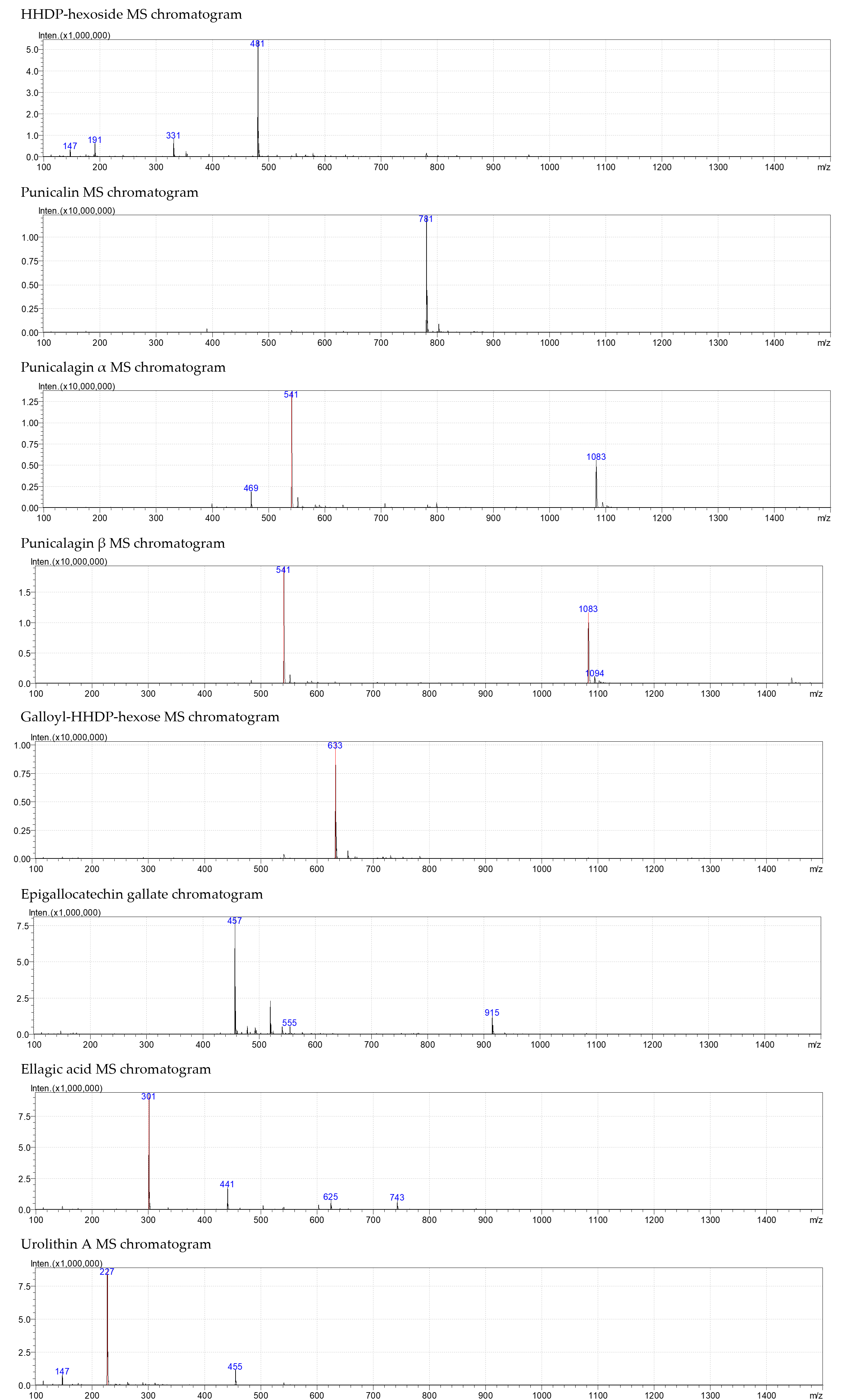


**Supplementary Figure 1**. MS chromatograms of each identified compound in APEx.


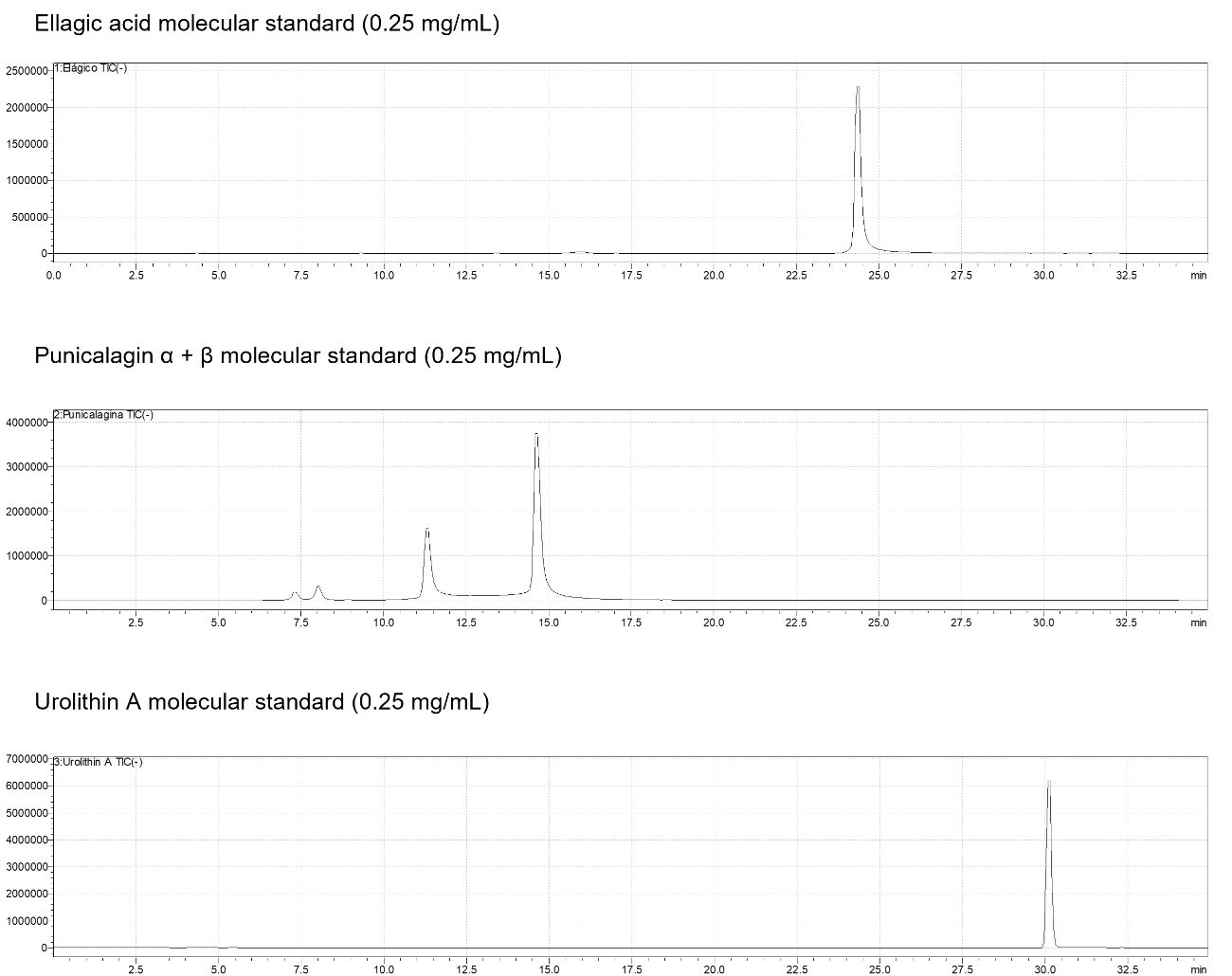


**Supplementary Figure 2**. Total ion chromatogram obtained by HPLC-MS of the molecular standards used to identify the main components of APEx.


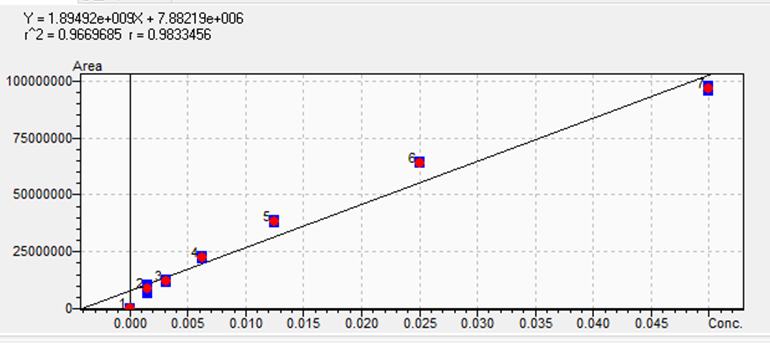


**Supplementary Figure 3.** Standard curve for urolithin A quantification obtained by HPLC-MS.
